# (2*E*,5*E*)-2,5-Bis(4-hy­droxy-3-meth­oxy­benzyl­idene)cyclo­penta­none ethanol monosolvate

**DOI:** 10.1107/S1600536813005229

**Published:** 2013-03-13

**Authors:** Muhammad Da’i, Arry Yanuar, Edy Meiyanto, Umar Anggara Jenie, Amir Margono Supardjan

**Affiliations:** a Faculty of Pharmacy, Muhammadiyah University, Jl. A. Yani Tromol Pos I Pabelan, Kartosuro, Surakarta 57162, Indonesia; b Faculty of Pharmacy, University of Indonesia, Kampus Universitas Indonesia, Depok 16424, Indonesia; c Faculty of Pharmacy, Gadjah Mada University, Sekip Utara, Yogyakarta 55281, Indonesia

## Abstract

In the title structure, C_21_H_20_O_5_·C_2_H_5_OH, the curcumine-type mol­ecule has a double *E* conformation for the two benzyl­idene double bonds [C=C = 1.342 (4) and 1.349 (4) Å] and is nearly planar with respect to the non-H atoms (r.m.s. deviation from planarity = 0.069 Å). The two phenolic OH groups form bifurcated hydrogen bonds with intra­molecular branches to adjacent meth­oxy O atoms and inter­molecular branches to either a neighbouring mol­ecule or an ethanol solvent mol­ecule. The ethanol O atom donates a hydrogen bond to the keto O atom. These hydrogen bonds link the constituents into layers parallel to (101) in the crystal structure.

## Related literature

For the biological activity of curcumin-type compounds, see: Ohori *et al.* (2006 ▶); Da’i *et al.* (2007 ▶); Anand *et al.* (2008 ▶). For the synthesis of the title compound, see: Sardjiman *et al.* (1997 ▶). For related structures, see: Du *et al.* (2010 ▶, 2011 ▶).
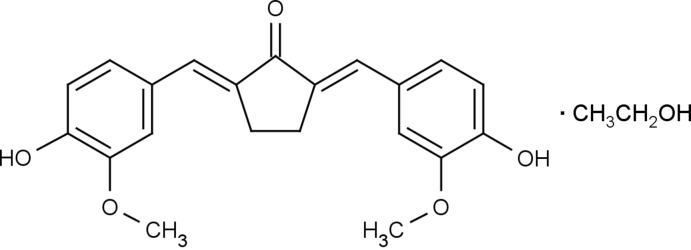


## Experimental

### 

#### Crystal data

C_21_H_20_O_5_·C_2_H_6_O
*M* *_r_* = 398.45
Monoclinic, 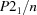

*a* = 8.880 (4) Å
*b* = 17.050 (5) Å
*c* = 13.950 (5) Å
β = 103.527 (14)°
*V* = 2053.4 (13) Å^3^
*Z* = 4
Mo *K*α radiation
μ = 0.09 mm^−1^
*T* = 296 K
0.12 × 0.10 × 0.06 mm

#### Data collection

Rigaku R-AXIS RAPID diffractometer
Absorption correction: multi-scan (*ABSCOR*; Rigaku, 1995 ▶) *T*_min_ = 0.671, *T*_max_ = 0.994
16083 measured reflections
3720 independent reflections
1748 reflections with *I* > 2σ(*I*)
*R*_int_ = 0.119

#### Refinement

*R*[*F* ^2^ > 2σ(*F* ^2^)] = 0.070
*wR*(*F* ^2^) = 0.157
*S* = 1.00
3720 reflections
268 parameters
H-atom parameters constrained
Δρ_max_ = 0.14 e Å^−3^
Δρ_min_ = −0.21 e Å^−3^

### 

Data collection: *RAPID-AUTO* (Rigaku, 2006 ▶); cell refinement: *RAPID-AUTO*; data reduction: *RAPID-AUTO*; program(s) used to solve structure: *SIR2008* (Burla *et al.*, 2007 ▶); program(s) used to refine structure: *SHELXL97* (Sheldrick, 2008 ▶); molecular graphics: *CrystalStructure* (Rigaku, 2010 ▶); software used to prepare material for publication: *publCIF* (Westrip, 2010 ▶).

## Supplementary Material

Crystal structure: contains datablock(s) global, I. DOI: 10.1107/S1600536813005229/qk2050sup1.cif

Structure factors: contains datablock(s) I. DOI: 10.1107/S1600536813005229/qk2050Isup2.hkl

Supplementary material file. DOI: 10.1107/S1600536813005229/qk2050Isup3.mol

Supplementary material file. DOI: 10.1107/S1600536813005229/qk2050Isup4.cml

Additional supplementary materials:  crystallographic information; 3D view; checkCIF report

## Figures and Tables

**Table 1:** Hydrogen-bond geometry (Å, °)

*D*—H⋯*A*	*D*—H	H⋯*A*	*D*⋯*A*	*D*—H⋯*A*
O3—H12⋯O2	0.82	2.21	2.653 (4)	114
O3—H12⋯O5^i^	0.82	2.13	2.802 (3)	139
O5—H20⋯O4	0.82	2.22	2.666 (3)	114
O5—H20⋯O6^ii^	0.82	1.93	2.682 (4)	151
O6—H26⋯O1^iii^	0.82	1.99	2.802 (4)	172

Symmetry codes: (i) 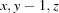; (ii) 
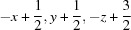
; (iii) 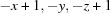.

**Fig. 1.**
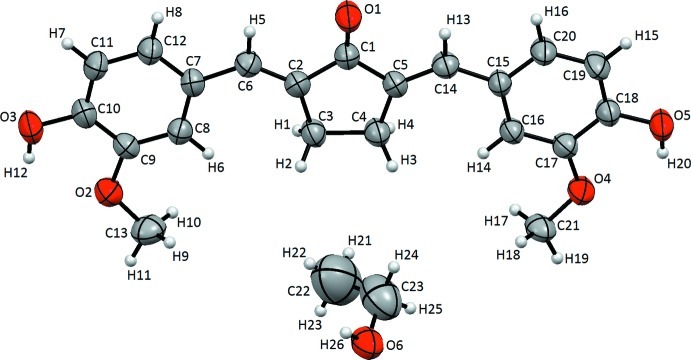
The asymmetric unit of (I), with the atomic numbering scheme. The thermal ellipsoids are scaled to 50% probability level.

**Fig. 2.**
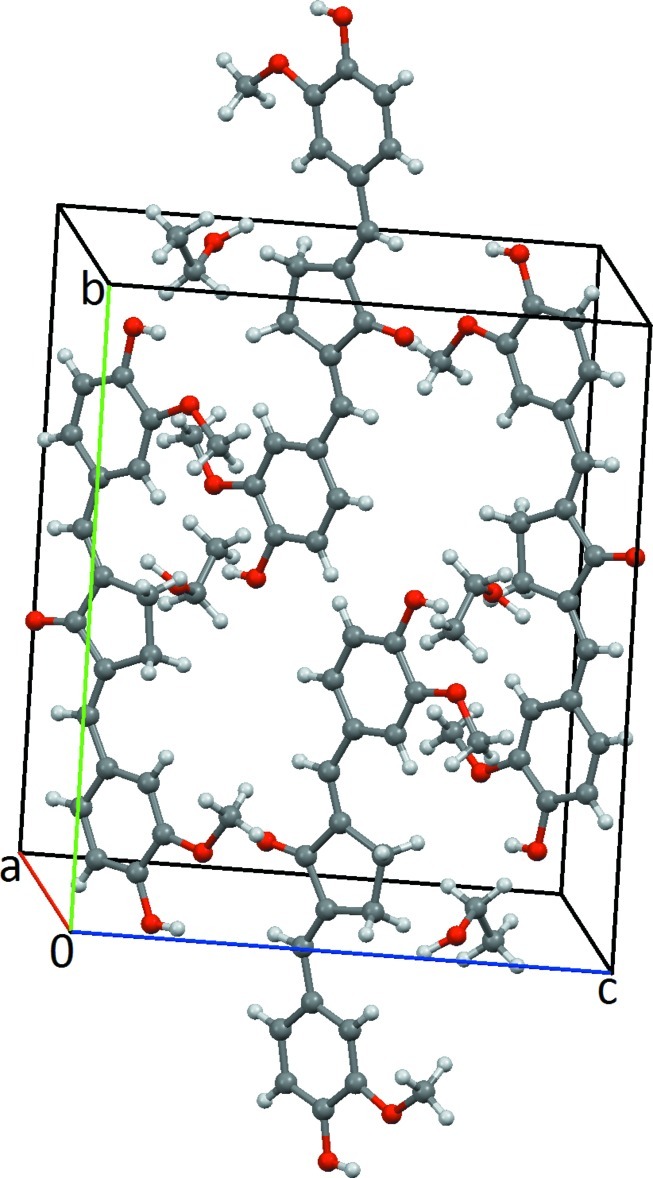
Crystal packing of the title compound.

## supplementary crystallographic information

### Comment

The title structure contains a curcumin analogue synthesized from vanillin (4-hydroxy-3-methoxybenzaldehyde) and cyclopentanone using acidic catalysis (Sardjiman *et al.*, 1997). This curcumin analogue showed good activities as anticancer agent in T47D cell line tests, and has also antioxidant and anti-inflammatory properties (Ohori *et al.*, 2006; Da'i *et al.*, 2007; Anand *et al.*, 2008). As an extension of the work on this compound (Sardjiman *et al.*, 1997; Da'i *et al.*, 2007) we report here the crystal structure of (I), an ethanol solvate. The curcumin type molecule contains two double bonds between C2 and C6 and C5 and C14 which connect the cyclopentanone fragment to the 4-hydroxy-3-methoxybenzylidene groups in *E* configuration relative to the carbonyl group. The entire molecule is essentially planar with respect to non-hydrogen atoms. The keto-diene system shows usual bond distances and angles. While the angles C1—C2=C6 = 118.9 (3)° and C1—C5=C14 = 118.7 (3)° are close to the ideal C_sp2_ bond angle of 120°, the bond angles C2=C6—C7 = 132.3 (3)° and C5=C14—C15 = 132.5 (3)° are large in response to intramolecular H···H contacts (phenyl H6 and H14 with the C_2_H_4_ group of the cyclopentanone ring). A partial conjugation between the C2=C6 and C5=C14 double bonds and the carbonyl group C1=O1 is evident from the bond lengths table. All other bond lengths and angles adopt usual values (Du *et al.*, 2010, 2011). In the crystal structure the curcumin type molecules are oriented approximately parallel to (203) and form together with the ethanol solvent molecules layer-like assemblies parallel to (101) linked via hydrogen bonds (Table 1). The two phenolic OH groups form bifurcated hydrogen bonds with intramolecular branches to adjacent methoxy O atoms (O3—H12···O5, O5—H20···O4) and intermolecular branches to either a neigbour molecule (O3—H12···O5^i^) or an ethanol molecule (O5—H20···O6^ii^). Ethanol donates a hydrogen bond to the keto-oxygen atom.

### Experimental

The compound was synthesized according to Sardjiman *et al.* (1997). It was then dissolved in boiling ethanol and crystallized by slow cooling giving yellowish crystals that were stored in cold ethanol prior to X-ray analysis.

### Refinement

All H atoms were placed in geometrically idealized positions (C—H = 0.93 – 0.97 Å, O—H = 0.82 Å) and constrained to ride on their parent atoms, with *U*_ĩso_(H) = 1.2*U*_eq_(C_sp2_) or 1.5*U*_eq_(C_sp3_,O). CH_3_ and OH groups were refined in orientation using AFIX 137 and AFIX 147 of program *SHELXL97* (Sheldrick, 2008).

### Crystal data

**Table:**

C_21_H_20_O_5_·C_2_H_6_O	*F*(000) = 848.00
*M**_r_* = 398.45	*D*_x_ = 1.289 Mg m^−^^3^
Monoclinic, *P*2_1_/*n*	Mo *K*α radiation, λ = 0.71075 Å
Hall symbol: -P 2yn	Cell parameters from 7488 reflections
*a* = 8.880 (4) Å	θ = 3.0–25.3°
*b* = 17.050 (5) Å	µ = 0.09 mm^−^^1^
*c* = 13.950 (5) Å	*T* = 296 K
β = 103.527 (14)°	Block, yellow
*V* = 2053.4 (13) Å^3^	0.12 × 0.10 × 0.06 mm
*Z* = 4	

### Data collection

**Table:**

Rigaku R-AXIS RAPID diffractometer	3720 independent reflections
Radiation source: fine-focus sealed tube	1748 reflections with *I* > 2σ(*I*)
Graphite monochromator	*R*_int_ = 0.119
ω scans	θ_max_ = 25.4°
Absorption correction: multi-scan (*ABSCOR*; Rigaku, 1995)	*h* = −10→10
*T*_min_ = 0.671, *T*_max_ = 0.994	*k* = −19→20
16083 measured reflections	*l* = −16→13

### Refinement

**Table:**

Refinement on *F*^2^	Primary atom site location: structure-invariant direct methods
Least-squares matrix: full	Secondary atom site location: difference Fourier map
*R*[*F*^2^ > 2σ(*F*^2^)] = 0.070	Hydrogen site location: inferred from neighbouring sites
*wR*(*F*^2^) = 0.157	H-atom parameters constrained
*S* = 1.00	*w* = 1/[σ^2^(*F*_o_^2^) + (0.0566*P*)^2^ + 0.4936*P*] where *P* = (*F*_o_^2^ + 2*F*_c_^2^)/3
3720 reflections	(Δ/σ)_max_ < 0.001
268 parameters	Δρ_max_ = 0.14 e Å^−^^3^
0 restraints	Δρ_min_ = −0.21 e Å^−^^3^

### Special details

**Table:**

Refinement. Refinement was performed using all reflections. The weighted *R*-factor (*wR*) and goodness of fit (*S*) are based on *F*^2^. *R*-factor (gt) are based on *F*. The threshold expression of *F*^2^ > 2.0 σ(*F*^2^) is used only for calculating *R*-factor (gt).

### Fractional atomic coordinates and isotropic or equivalent isotropic displacement parameters (Å^2^)

**Table:**

	*x*	*y*	*z*	*U*_iso_*/*U*_eq_	
O1	0.8369 (3)	0.06512 (12)	0.4353 (2)	0.0667 (8)	
O2	0.5966 (3)	−0.31177 (12)	0.67112 (19)	0.0615 (8)	
O3	0.7414 (4)	−0.40870 (12)	0.5739 (2)	0.0710 (8)	
H12	0.6808	−0.4176	0.6088	0.107*	
O4	0.4611 (3)	0.35840 (12)	0.73021 (19)	0.0604 (8)	
O5	0.5611 (3)	0.48579 (11)	0.65321 (19)	0.0633 (8)	
H20	0.5129	0.4816	0.6964	0.095*	
C1	0.7723 (4)	0.05470 (17)	0.5029 (3)	0.0476 (9)	
C2	0.7402 (4)	−0.02157 (17)	0.5437 (2)	0.0445 (9)	
C3	0.6494 (4)	−0.00895 (16)	0.6200 (3)	0.0486 (9)	
H1	0.7029	−0.0318	0.6824	0.058*	
H2	0.5480	−0.0330	0.5996	0.058*	
C4	0.6338 (4)	0.08115 (17)	0.6307 (3)	0.0503 (10)	
H3	0.5256	0.0963	0.6174	0.060*	
H4	0.6845	0.0980	0.6967	0.060*	
C5	0.7110 (4)	0.11694 (17)	0.5560 (2)	0.0437 (9)	
C6	0.7921 (4)	−0.08723 (17)	0.5092 (3)	0.0479 (9)	
H5	0.8498	−0.0782	0.4626	0.057*	
C7	0.7754 (4)	−0.16971 (17)	0.5307 (2)	0.0444 (9)	
C8	0.6881 (4)	−0.19778 (17)	0.5944 (2)	0.0459 (9)	
H6	0.6365	−0.1626	0.6265	0.055*	
C9	0.6773 (4)	−0.27714 (18)	0.6102 (3)	0.0467 (9)	
C10	0.7508 (4)	−0.33004 (18)	0.5607 (3)	0.0501 (10)	
C11	0.8375 (5)	−0.30373 (18)	0.4982 (3)	0.0548 (10)	
H7	0.8880	−0.3392	0.4660	0.066*	
C12	0.8498 (4)	−0.22375 (17)	0.4831 (3)	0.0521 (10)	
H8	0.9088	−0.2060	0.4404	0.062*	
C13	0.5081 (5)	−0.2631 (2)	0.7187 (3)	0.0695 (12)	
H9	0.4335	−0.2347	0.6703	0.104*	
H10	0.5753	−0.2268	0.7609	0.104*	
H11	0.4554	−0.2948	0.7575	0.104*	
C14	0.7297 (4)	0.19233 (17)	0.5318 (2)	0.0469 (9)	
H13	0.7838	0.1983	0.4826	0.056*	
C15	0.6821 (4)	0.26566 (16)	0.5675 (2)	0.0429 (9)	
C16	0.5905 (4)	0.27266 (17)	0.6358 (2)	0.0441 (9)	
H14	0.5571	0.2276	0.6622	0.053*	
C17	0.5488 (4)	0.34504 (18)	0.6647 (2)	0.0463 (9)	
C18	0.5994 (4)	0.41270 (18)	0.6257 (3)	0.0489 (9)	
C19	0.6884 (5)	0.40735 (19)	0.5584 (3)	0.0588 (11)	
H15	0.7217	0.4526	0.5323	0.071*	
C20	0.7290 (4)	0.33428 (18)	0.5292 (3)	0.0541 (10)	
H16	0.7891	0.3310	0.4829	0.065*	
C21	0.3987 (4)	0.29279 (19)	0.7699 (3)	0.0586 (11)	
H17	0.4815	0.2599	0.8044	0.088*	
H18	0.3334	0.2636	0.7175	0.088*	
H19	0.3389	0.3105	0.8149	0.088*	
O6	0.1141 (4)	0.02592 (15)	0.7218 (2)	0.0752 (9)	
H26	0.1194	−0.0016	0.6746	0.113*	
C22	0.3617 (9)	−0.0071 (4)	0.8151 (6)	0.172 (3)	
H21	0.4563	0.0143	0.8541	0.257*	
H22	0.3847	−0.0413	0.7657	0.257*	
H23	0.3098	−0.0362	0.8568	0.257*	
C23	0.2672 (9)	0.0531 (3)	0.7700 (5)	0.122 (2)	
H24	0.3141	0.0781	0.7217	0.147*	
H25	0.2586	0.0921	0.8191	0.147*	

### Atomic displacement parameters (Å^2^)

**Table:**

	*U*^11^	*U*^22^	*U*^33^	*U*^12^	*U*^13^	*U*^23^
O1	0.094 (2)	0.0421 (14)	0.080 (2)	−0.0005 (13)	0.0528 (19)	−0.0005 (12)
O2	0.069 (2)	0.0486 (14)	0.0746 (19)	0.0009 (13)	0.0332 (16)	0.0090 (12)
O3	0.092 (2)	0.0348 (14)	0.097 (2)	−0.0040 (13)	0.0435 (17)	0.0012 (12)
O4	0.079 (2)	0.0435 (14)	0.0726 (19)	−0.0005 (12)	0.0454 (17)	0.0038 (12)
O5	0.090 (2)	0.0348 (13)	0.078 (2)	0.0002 (12)	0.0455 (17)	−0.0016 (11)
C1	0.052 (3)	0.037 (2)	0.057 (3)	−0.0001 (16)	0.019 (2)	−0.0001 (17)
C2	0.043 (2)	0.040 (2)	0.051 (2)	−0.0009 (15)	0.013 (2)	−0.0001 (16)
C3	0.056 (3)	0.0378 (18)	0.053 (2)	−0.0033 (16)	0.015 (2)	0.0043 (15)
C4	0.061 (3)	0.0409 (19)	0.054 (2)	−0.0044 (17)	0.022 (2)	−0.0033 (16)
C5	0.046 (2)	0.0357 (19)	0.051 (2)	−0.0039 (15)	0.0140 (19)	0.0004 (15)
C6	0.053 (3)	0.043 (2)	0.052 (2)	0.0002 (17)	0.020 (2)	0.0017 (16)
C7	0.049 (3)	0.0351 (19)	0.050 (2)	0.0004 (16)	0.012 (2)	0.0022 (15)
C8	0.049 (3)	0.040 (2)	0.051 (2)	0.0038 (16)	0.015 (2)	0.0010 (16)
C9	0.049 (3)	0.041 (2)	0.053 (2)	−0.0049 (16)	0.017 (2)	0.0039 (16)
C10	0.055 (3)	0.036 (2)	0.059 (3)	−0.0047 (17)	0.014 (2)	0.0006 (17)
C11	0.063 (3)	0.039 (2)	0.067 (3)	0.0010 (17)	0.026 (2)	−0.0100 (17)
C12	0.061 (3)	0.039 (2)	0.060 (3)	0.0009 (17)	0.023 (2)	−0.0033 (16)
C13	0.070 (3)	0.078 (3)	0.070 (3)	0.007 (2)	0.034 (3)	0.006 (2)
C14	0.054 (3)	0.046 (2)	0.044 (2)	−0.0016 (17)	0.018 (2)	−0.0015 (16)
C15	0.051 (3)	0.0325 (18)	0.048 (2)	−0.0010 (15)	0.016 (2)	0.0000 (15)
C16	0.052 (3)	0.0359 (19)	0.048 (2)	−0.0040 (16)	0.018 (2)	0.0014 (15)
C17	0.050 (3)	0.042 (2)	0.049 (2)	−0.0051 (16)	0.018 (2)	−0.0027 (16)
C18	0.059 (3)	0.037 (2)	0.054 (2)	−0.0043 (17)	0.020 (2)	−0.0039 (16)
C19	0.079 (3)	0.038 (2)	0.071 (3)	−0.0042 (19)	0.042 (3)	0.0082 (18)
C20	0.072 (3)	0.041 (2)	0.060 (3)	−0.0007 (18)	0.037 (2)	0.0005 (17)
C21	0.068 (3)	0.058 (2)	0.058 (3)	−0.0111 (19)	0.033 (2)	−0.0002 (18)
O6	0.092 (3)	0.0635 (18)	0.082 (2)	−0.0124 (16)	0.0453 (19)	−0.0068 (14)
C22	0.151 (7)	0.157 (6)	0.201 (8)	0.001 (5)	0.030 (6)	0.041 (6)
C23	0.158 (7)	0.105 (4)	0.113 (5)	−0.026 (4)	0.051 (5)	0.003 (4)

### Geometric parameters (Å, º)

**Table:**

O1—C1	1.226 (4)	C11—C12	1.388 (4)
O2—C9	1.368 (4)	C11—H7	0.9300
O2—C13	1.411 (4)	C12—H8	0.9300
O3—C10	1.359 (4)	C13—H9	0.9600
O3—H12	0.8200	C13—H10	0.9600
O4—C17	1.351 (4)	C13—H11	0.9600
O4—C21	1.417 (4)	C14—C15	1.445 (4)
O5—C18	1.370 (4)	C14—H13	0.9300
O5—H20	0.8200	C15—C20	1.390 (4)
C1—C5	1.469 (4)	C15—C16	1.395 (4)
C1—C2	1.474 (4)	C16—C17	1.376 (4)
C2—C6	1.342 (4)	C16—H14	0.9300
C2—C3	1.493 (4)	C17—C18	1.394 (4)
C3—C4	1.553 (4)	C18—C19	1.365 (5)
C3—H1	0.9700	C19—C20	1.385 (4)
C3—H2	0.9700	C19—H15	0.9300
C4—C5	1.504 (5)	C20—H16	0.9300
C4—H3	0.9700	C21—H17	0.9600
C4—H4	0.9700	C21—H18	0.9600
C5—C14	1.349 (4)	C21—H19	0.9600
C6—C7	1.453 (4)	O6—C23	1.445 (7)
C6—H5	0.9300	O6—H26	0.8200
C7—C12	1.390 (4)	C22—C23	1.382 (7)
C7—C8	1.394 (4)	C22—H21	0.9600
C8—C9	1.378 (4)	C22—H22	0.9600
C8—H6	0.9300	C22—H23	0.9600
C9—C10	1.388 (5)	C23—H24	0.9700
C10—C11	1.366 (5)	C23—H25	0.9700
			
C9—O2—C13	118.0 (3)	O2—C13—H9	109.5
C10—O3—H12	109.5	O2—C13—H10	109.5
C17—O4—C21	118.1 (2)	H9—C13—H10	109.5
C18—O5—H20	109.5	O2—C13—H11	109.5
O1—C1—C5	125.3 (3)	H9—C13—H11	109.5
O1—C1—C2	126.3 (3)	H10—C13—H11	109.5
C5—C1—C2	108.4 (3)	C5—C14—C15	132.5 (3)
C6—C2—C1	118.9 (3)	C5—C14—H13	113.8
C6—C2—C3	131.6 (3)	C15—C14—H13	113.8
C1—C2—C3	109.5 (3)	C20—C15—C16	117.7 (3)
C2—C3—C4	106.6 (3)	C20—C15—C14	117.2 (3)
C2—C3—H1	110.4	C16—C15—C14	125.0 (3)
C4—C3—H1	110.4	C17—C16—C15	121.2 (3)
C2—C3—H2	110.4	C17—C16—H14	119.4
C4—C3—H2	110.4	C15—C16—H14	119.4
H1—C3—H2	108.6	O4—C17—C16	125.9 (3)
C5—C4—C3	105.6 (3)	O4—C17—C18	114.4 (3)
C5—C4—H3	110.6	C16—C17—C18	119.6 (3)
C3—C4—H3	110.6	C19—C18—O5	118.4 (3)
C5—C4—H4	110.6	C19—C18—C17	120.3 (3)
C3—C4—H4	110.6	O5—C18—C17	121.3 (3)
H3—C4—H4	108.7	C18—C19—C20	119.7 (3)
C14—C5—C1	118.7 (3)	C18—C19—H15	120.2
C14—C5—C4	131.5 (3)	C20—C19—H15	120.2
C1—C5—C4	109.8 (3)	C19—C20—C15	121.5 (3)
C2—C6—C7	132.3 (3)	C19—C20—H16	119.3
C2—C6—H5	113.8	C15—C20—H16	119.3
C7—C6—H5	113.8	O4—C21—H17	109.5
C12—C7—C8	118.3 (3)	O4—C21—H18	109.5
C12—C7—C6	117.4 (3)	H17—C21—H18	109.5
C8—C7—C6	124.3 (3)	O4—C21—H19	109.5
C9—C8—C7	120.6 (3)	H17—C21—H19	109.5
C9—C8—H6	119.7	H18—C21—H19	109.5
C7—C8—H6	119.7	C23—O6—H26	109.5
O2—C9—C8	126.1 (3)	C23—C22—H21	109.5
O2—C9—C10	113.8 (3)	C23—C22—H22	109.5
C8—C9—C10	120.1 (3)	H21—C22—H22	109.5
O3—C10—C11	118.2 (3)	C23—C22—H23	109.5
O3—C10—C9	121.5 (3)	H21—C22—H23	109.5
C11—C10—C9	120.3 (3)	H22—C22—H23	109.5
C10—C11—C12	119.7 (3)	C22—C23—O6	112.2 (5)
C10—C11—H7	120.1	C22—C23—H24	109.2
C12—C11—H7	120.1	O6—C23—H24	109.2
C11—C12—C7	121.0 (3)	C22—C23—H25	109.2
C11—C12—H8	119.5	O6—C23—H25	109.2
C7—C12—H8	119.5	H24—C23—H25	107.9
			
O1—C1—C2—C6	3.9 (6)	C8—C9—C10—C11	1.8 (6)
C5—C1—C2—C6	−177.1 (3)	O3—C10—C11—C12	−179.9 (3)
O1—C1—C2—C3	−175.8 (4)	C9—C10—C11—C12	−1.1 (6)
C5—C1—C2—C3	3.2 (4)	C10—C11—C12—C7	0.1 (6)
C6—C2—C3—C4	176.9 (4)	C8—C7—C12—C11	0.1 (5)
C1—C2—C3—C4	−3.5 (4)	C6—C7—C12—C11	−178.9 (3)
C2—C3—C4—C5	2.5 (4)	C1—C5—C14—C15	178.7 (4)
O1—C1—C5—C14	−2.1 (6)	C4—C5—C14—C15	−0.6 (7)
C2—C1—C5—C14	178.9 (3)	C5—C14—C15—C20	176.5 (4)
O1—C1—C5—C4	177.4 (4)	C5—C14—C15—C16	−4.7 (6)
C2—C1—C5—C4	−1.6 (4)	C20—C15—C16—C17	−0.4 (5)
C3—C4—C5—C14	178.8 (4)	C14—C15—C16—C17	−179.1 (3)
C3—C4—C5—C1	−0.6 (4)	C21—O4—C17—C16	−3.4 (5)
C1—C2—C6—C7	−177.4 (3)	C21—O4—C17—C18	177.1 (3)
C3—C2—C6—C7	2.2 (7)	C15—C16—C17—O4	−179.9 (3)
C2—C6—C7—C12	−177.9 (4)	C15—C16—C17—C18	−0.4 (5)
C2—C6—C7—C8	3.2 (6)	O4—C17—C18—C19	−179.8 (3)
C12—C7—C8—C9	0.6 (5)	C16—C17—C18—C19	0.7 (6)
C6—C7—C8—C9	179.5 (3)	O4—C17—C18—O5	0.0 (5)
C13—O2—C9—C8	3.8 (5)	C16—C17—C18—O5	−179.6 (3)
C13—O2—C9—C10	−175.2 (3)	O5—C18—C19—C20	180.0 (3)
C7—C8—C9—O2	179.5 (3)	C17—C18—C19—C20	−0.3 (6)
C7—C8—C9—C10	−1.6 (5)	C18—C19—C20—C15	−0.5 (6)
O2—C9—C10—O3	−0.4 (5)	C16—C15—C20—C19	0.8 (5)
C8—C9—C10—O3	−179.4 (3)	C14—C15—C20—C19	179.6 (3)
O2—C9—C10—C11	−179.1 (3)		

### Hydrogen-bond geometry (Å, º)

**Table:**

*D*—H···*A*	*D*—H	H···*A*	*D*···*A*	*D*—H···*A*
O3—H12···O2	0.82	2.21	2.653 (4)	114
O3—H12···O5^i^	0.82	2.13	2.802 (3)	139
O5—H20···O4	0.82	2.22	2.666 (3)	114
O5—H20···O6^ii^	0.82	1.93	2.682 (4)	151
O6—H26···O1^iii^	0.82	1.99	2.802 (4)	172

Symmetry codes: (i) *x*, *y*−1, *z*; (ii) −*x*+1/2, *y*+1/2, −*z*+3/2; (iii) −*x*+1, −*y*, −*z*+1.
